# Characterization and comparative DNA methylation profiling of four adipogenic genes in adipose-derived stem cells and dedifferentiated fat cells from aging subjects

**DOI:** 10.1007/s13577-020-00379-x

**Published:** 2020-06-03

**Authors:** Kallapat Tansriratanawong, Isao Tabei, Hiroshi Ishikawa, Akihiro Ohyama, Junko Toyomura, Soh Sato

**Affiliations:** 1 Department of Oral Medicine and Periodontology, Faculty of Dentistry, Mahidol University, 6 Yothi Street Rajthevi, Bangkok, 10400 Thailand; 2 Department of Surgery, Jikei University School of Medicine, Tokyo, 105-0003 Japan; 3 Department of Neurosurgery, Faculty of Medicine, University of Tsukuba, Ibaraki, 305-8575 Japan; 4 Department of Periodontology, Nippon Dental University, Niigata, 951-1500 Japan

**Keywords:** Adipose derived stem cells, DFAT cells, DNA methylation, Aging, Characterization, Epigenetics

## Abstract

Adipose-derived stem cells (ASCs) and dedifferentiated fat (DFAT) cells are alternative cell sources in tissue engineering and regeneration because they are easily obtained and exhibit multilineage differentiation. However, aging may attenuate their regenerative potential and metabolic functions. Reports characterizing DFAT cells derived from aging donors are rare, and comparisons of DNA methylation profiles between aging ASCs and DFAT cells are poorly understood. Therefore, this study aimed to characterize DFAT cells relative to ASCs derived from aging subjects and compare the DNA methylation profiles of four adipogenic genes in these cells. ASCs and DFAT cells from aging donors exhibited characteristics similar to those of stem cells, including colony formation, proliferation, and multilineage differentiation abilities. However, compared with ASCs, DFAT cells exhibited increased proliferation, smooth muscle actin alpha (SMA-α) expression and decreased cellular senescence. DNA methylation profiling of ASCs and DFAT cells by combined bisulfite restriction analysis (COBRA) demonstrated hypermethylation patterns in three potent adipogenic genes—peroxisome proliferator-activated receptor gamma 2 (PPARγ2), fatty acid-binding protein 4 (FABP4), and lipoprotein lipase (LPL)—but hypomethylation of CCAAT/enhancer binding protein alpha (C/EBPα) in the aging group. Statistically significant differences were observed between the aging group and the young group. Epigenetic regulation maintains the stability of ASCs and DFAT cells in an age-dependent manner. Our findings suggested that although the DNA methylation patterns of three adipogenic genes correlated with hypermethylation and aging, ASCs and DFAT cells exhibited cellular stability and several stem cell characteristics, offering further opportunities for personalized regeneration and energy maintenance by adipogenesis during aging.

**Electronic supplementary material:**

The online version of this article (10.1007/s13577-020-00379-x) contains supplementary material, which is available to authorized users.

## Introduction

Adipose-derived stem cells (ASCs) are a source of multipotent mesenchymal stem cells (MSCs) for tissue engineering and regeneration [1, 2]. ASCs are consistent with the definition of stem cells; they exhibit clonality, proliferation, multilineage differentiation, and long-term culture expansion with the same efficacy as bone marrow mesenchymal stem cells (BMMSCs) [3–7]. Moreover, large numbers of ASCs can be obtained via noninvasive techniques, and ASCs are less ethically controversial than embryonic stem cells. However, their heterogeneity is reflected in variations in plasticity, proliferation, and differentiation between ASCs obtained from distinct adipose tissue depots or even from self-clones [8, 9]. Their surface immunophenotypes show expression of various CD antigen markers possibly derived from stromal vascular (CD31, CD34, CD45, and CD146 [10, 11]) or mesenchymal (CD13, CD29, CD44, CD73, CD90, and CD105 [12–15]) populations. In addition to ASCs, dedifferentiated fat cells (DFAT cells) have been recently indicated for use in tissue engineering [16, 17]. They are derived from mature adipocytes, which exhibit cellular homogeneity, and can transdifferentiate into several mature cell types, including osteoblasts [18], cardiocytes [19], and urethral sphincter cells [20]. However, much more is known about the proliferation, immunophenotypic, and differentiation characteristics of ASCs than those of DFAT cells.

Epigenetic traits are transcriptional and translational changes invoked by chemical modifications to nucleotides and proteins without alteration of the original sequences [21, 22]. Epigenetic modifications affect several cellular processes, including proliferation and differentiation, and are divided into two major classes: DNA methylation and histone modification. Although DNA methylation has been widely investigated, particularly in cancer research, research on methylation in stem cells is rare. DNA methylation is believed to mediate tissue homeostasis, long-term gene silencing, X chromosome inactivation, and genomic imprinting [22–26]. Furthermore, DNA methylation can be influenced by the environment, patient factors, and aging [24, 25].

Several studies using MSCs from aging donors have produced conflicting results as to whether cellular processes are slowed [27, 28]. Generally, MSCs aging can be classified as in vitro aging (cellular senescence), which arises from cell passaging, or in vivo aging, occurring in cells derived from aging donors. Aging is defined as the accumulation of changes in cells and tissues that increase the risk of disease and death [29]. Several theories have been proposed to explain the foremost causes of aging, for example, the free radical theory [29], mitochondrial theory [30], and inflammation theory [31]. Recently, the role of epigenetics in aging was reported as the identification of genome-wide and locus-specific differences by cytosine methylation analysis. Genome-wide analysis studies of aging adipose cells have revealed only slight changes or stability in DNA methylation in the adipogenic differentiation stage, indicating that the methylation status in adipose tissue is stable with age, in contrast to the pattern observed in other tissues, such as liver [25, 32, 33]. However, DNA methylation has been shown to increase with advancing age [34, 35], and changes in DNA methylation during pluripotent stem cell induction have been observed [36]. Also, the anatomical location of the examined cells, and the site specificity of the CpG content in each gene might result from the dynamics of epigenetic drift.

Epigenetic regulation plays several pivotal roles in adipose cells of aging people. They can be used for screening in regenerative medicine to easily select as cellular sources and to improve the cellular defects associated with aging and age-related diseases, such as diabetes mellitus, cardiovascular disease, and cancer. Epigenetic drugs have been developed to target epigenetic defects in various diseases, and the development of epigenetic therapeutics is increasing, as demonstrated by DNA methyltransferase (DNMT) inhibitors, decitabine and histone deacetylase (HDAC) inhibitors, which are widely used for cancer therapy [37, 38]. Epigenetic changes might mediate cellular rejuvenation, which can re-establish stem cell pluripotency and prolong the life span of aging people. Furthermore, adipogenicity maintains energy metabolism and facilitates appropriate energy expenditure during aging processes; thus, our study attempted to clarify that epigenetics regulations may give informative data and prove that ASCs and DFAT cells might be appropriate sources for regeneration in aging individuals. However, the relationship of adipogenic cells to aging and epigenetic regulation remains unclear, and studies on DNA methylation in ASCs and DFAT cells are rare and demonstrate inconclusive results [8, 21]. We examined the proximal promoters of four adipogenic genes identified as having significant roles in adipogenesis: peroxisome proliferator-activated receptor gamma 2 (PPARγ2), fatty acid-binding protein 4 (FABP4), lipoprotein lipase (LPL), and CCAAT/enhancer binding protein alpha (C/EBPα) [8]. Thus, this recent study hypothesized that DNA methylation profiles might have implications on cellular processes in ASCs and DFAT cells during aging.

Therefore, we first screened DFAT cells derived from aging individuals and investigated their characteristics to determine whether they exhibit features similar to those of aging ASCs. Then, DNA methylation was analyzed to determine its relationship to aging. We aimed to (1) compare the phenotypic characteristics of ASCs and DFAT cells from aging subjects and (2) examine the DNA methylation profiles of four putative adipogenic genes in early- and late-passage ASCs and DFAT cells from aging and young subjects to clarify the role of epigenetic regulation in adipose cells.

## Materials and methods

### Isolation and culture of ASCs and DFAT cells

The protocol was approved by the ethics committees of Nippon Dental University (NDU-T2011-32). All subjects provided written informed consent for participation in the study. Subcutaneous adipose tissues were isolated from total 21 aging subjects [6 men and 15 women, aged between 64 and 90 (77.33 ± 7) years] as discarded products from hip or thigh surgeries or liposuction, and sample from 25 young subjects [1 man and 24 women, aged between 20 and 37 (30.04 ± 4.58) years] were kindly provided by Dr. Isao Tabei from the Department of Surgery, Jikei University School of Medicine, Tokyo, Japan. ASCs were isolated and cultured as described earlier with slight modification [5]. Adipose tissues (1–5 g) were rinsed with Hanks’ balanced salt solution (HBSS; Gibco, USA) and minced into small pieces. The enzymatic digestion method of incubation with warmed collagenase type I (3 mg/mL; Sigma-Aldrich, St Louis, MO, USA) and dispase (4 mg/mL; Sanko Pure Chemical Ltd., Tokyo, Japan) at 37 °C for 1 h (h) was used to obtain primary cells. Cell suspensions were filtered through 70-µm cell strainers (Falcon, BD Labware, NJ, USA) and then centrifuged at 300×*g* for 15 min (min). The cell pellets at the bottom of the tube were called the stromal vascular fraction (SVF), which was subsequently used for ASCs culture. In contrast, the uppermost layer was collected for DFAT cells culture. Erythrocyte lysis buffer was added to those two fractions and incubated at 4 °C for 15 min, followed by centrifugation at 300×*g* for 5 min. SVF pellets were recovered by culture in growth medium (GM) at 37 ºC and 4.7% CO_2_. Dulbecco’s modified Eagle’s medium/Ham’s nutrient mixture F12 (DMEM/F12, Gibco BRL, CA, USA) supplemented with 15% fetal bovine serum (FBS, Gibco BRL), 2 mM glutamine (GlutaMAX I, Invitrogen, CA, USA), 50 U/mL penicillin and 50 μg/mL streptomycin (Gibco BRL) was used as GM for culture. Cells (1 × 10^4^ cells per 10-cm^2^ dish (Nunc, Roskilde, Denmark)) from the SVF were plated as primary culture. The uppermost fractionated layer was cultured as described earlier with slight modifications [16]. The uppermost layers of mature fat tissues were isolated in 25-cm^2^ culture flask (Nunc), which was completely filled with GM. Mature adipocytes floated and attached to the upper surface of the flask. The flask was then inverted after 7–10 days with a reduced (2/3) volume of GM. Cells cultured in this manner were subsequently referred to DFAT cells. At 80–90% confluence, ASCs and DFAT cells were harvested by the addition of 0.1% trypsin and 0.02% ethylenediaminetetraacetic acid (EDTA)/phosphate buffer saline (PBS) and split at a 1:3 ratio in fresh medium. Cells from passage (P) 3–8 were used in subsequent experiments. For BMMSCs, four cell lines of P3, which were kindly provided by Dr. Yuichi Tamaki (The Nippon Dental University School of Life Dentistry at Tokyo, Japan), were used as MSCs controls in RT-PCR of multilineage differentiation.

### Magnetic beads isolation

For isolating ASCs from endothelial cells, CD31 magnetic beads (Miltenyi Biotech, Bergish, Gladbach, Germany) and a miniMACs magnet kit (Miltenyl Biotech) were used [8]. Primary ASCs cultures (1 × 10^7^ cells) were trypsinized and centrifuged at 300×*g* for 5 min. Cells were then recovered in 60 μL of GM. After blocking from 20 μL of FcR blocking reagent, 20 μL of CD31 microbeads were added to the cells and incubated at 4 °C for 15 min. The microbeads were applied to the magnetic column. The unlabeled cells (CD31^−^) were collected, whereas the labeled cells (CD31^+^) were discarded. In this study, CD31^−^ ASCs were called ASCs, and subsequently seeded at 1 × 10^4^ cells per 60-mm^2^ dish (Falcon).

### Colony-forming unit (CFU) assay

Aging ASCs and DFAT cells (1 × 10^3^ cells/mL) were separately plated in 10-cm^2^ dishes (Nunc) and cultured in GM for 7 days. Then, cells were fixed with 10% formalin solution (Wako Pure Chemical Industries, Ltd, Japan) and stained with 0.1% toluidine Blue (Muto Pure Chemical, Japan) for 5 min. The aggregated colonies containing 50 or more cells and having a diameter exceeding 2 mm were counted under a phase contrast inverted microscope (Olympus Optical Co. Ltd, Tokyo, Japan). The CFU (%) was calculated as follow: number of colonies per plate/number of cells seeded × 100.

### MTT assay

3-(4,5-Dimethyl-2-thiazolyl)-2,5-diphenyl-2*H*-tetrazolium bromide (MTT; 5 mg/mL; Roche Applied Science) was used to assess the proliferative ability of aging ASCs and DFAT cells. Cells (1 × 10^3^ cells/well) were seeded in 24-well plate (Nunc) and incubated overnight. Then, 50 μL of MTT solution was added to each well and incubated for 4 h at 37 °C to allow formazan crystal formation. After incubation, the formazan crystals were dissolved in 500 μL of MTT solubilization solution and further incubated overnight. Optical density values at 570 nm were determined spectrophotometrically in a microplate reader (Viento, Bio-Tek®). The wells containing MTT but no seeded cells were used as the negative control.

### Population doubling time (PDT) assay

Aging ASCs and DFAT cells of P3 were seeded in triplicate at 1 × 10^3^ cells per 35-mm^2^ dish (Nunc). Cells were counted at 2-day intervals for 12 days. The PDT was calculated from the logarithmic phase of the growth curve using the following formula: PDT = duration × log 2/log (final concentration) − log (initial concentration).

### Flow cytometric analysis

For identification of immunophenotypes, aging ASCs and DFAT cells were harvested at P4. Cells were trypsinized, and viability was assessed using 0.4% Trypan Blue (Gibco) and found to be greater than 95%. Then, cells were separated into tubes containing 5 × 10^5^ cells each. The following mouse monoclonal anti-human antibodies conjugated with fluorescein isothiocyanate-conjugated (FITC) and phycoerythrin (PE) were used: anti-CD29-PE, anti-CD31-PE, anti-CD73-PE, anti-CD90-PE, anti-CD105-PE, anti-CD106-PE, anti-CD146-PE (all from BD Biosciences, CA, USA): anti-CD34-FITC, and anti-CD44-FITC (Beckman coulter Inc.); and immunoglobulin G1 isotype control (BD Biosciences). Each aliquot was incubated in the dark at 4 °C for 20 min. Then, cell pellets were washed with PBS and resuspended in 0.1% bovine serum albumin (BSA)/PBS. Flow cytometric data were analyzed with Guava Express Plus version 5.3 software (Guava Technology).

### Immunofluorescence

Aging ASCs and DFAT cells from at P4 were fixed with cold 100% methanol at 4 ºC for 10 min. Then, 1% BSA/PBS, was added as a blocking reagent at room temperature (25 ºC) for 30 min. Cells were incubated at 25 ºC for 2 h with the following primary antibodies: polyclonal rabbit anti-cytokeratin-14 (CK-14, 1:400; Dako Corporation), monoclonal mouse anti-mitochondria (1:200; Millipore, Bedford, MA), polyclonal rabbit anti-nestin (1:200; Millipore), monoclonal mouse anti-vimentin (1:200; Sigma), polyclonal rabbit anti-proliferating cell nuclear antigen (PCNA, 1:100; Santa cruz biotechnology), monoclonal mouse anti-smooth muscle actin alpha (SMA-α, 1:200; Millipore), and polyclonal rabbit anti-von Willebrand factor (vWF, 1:200; Abcam, Cambridge, UK). Then, cells were incubated with secondary antibodies, including Alexa Fluor 568-conjugated goat anti-mouse IgG and Alexa Fluor 488-conjugated donkey anti-rabbit, at a dilution of 1:500 for 30 min in the dark. All cells were counterstained and mounted with Vectashield mounting medium containing 4,6-diamidino-2-phenyl indole (DAPI) (Vector Laboratories, CA, USA). Images were acquired using a fluorescence microscope (BZ-9000, KEYENCE, Tokyo, Japan). Cells not incubated with the primary antibodies were used as negative control cells.

### Multilineage differentiation

For osteogenic and adipogenic differentiation, aging ASCs and DFAT cells at P3 were plated in a monolayer at a density of 1 × 10^4^ cells per well in 6-well plates (Nunc). Cells that reached to 80–90% confluence were then replaced with each differentiation medium. For osteogenic differentiation, the medium was supplemented with 100 nM dexamethasone (Sigma-Aldrich), 50 μM ascorbic acid (Sigma-Aldrich), and 10 mM β-glycerophosphate (Sigma-Aldrich) and cultured for 3 weeks. Then, differentiated cells were fixed with 10% formalin solution for 10 min and stained with 1% Alizarin Red (Certistain®, Darmstadt, Germany) at pH 4.2 for 30 min. For adipogenic differentiation, the medium was supplemented with 1 µM dexamethasone (Sigma-Aldrich), 0.5 mM isobutylmethylxanthine (IBMX; Sigma-Aldrich), and 100 μM indomethacin (Sigma-Aldrich) for 3 weeks. Then, cells were fixed with 10% formalin solution for 10 min and incubated in 60% isopropanol for 5 min. Oil Red O (Wako) was added and incubated for 15 min to stain lipid droplets. For chondrogenic differentiation, a three-dimensional (3D) culture system was used. In brief, approximately 1 × 10^7^ cells in 1 mL chondrogenic differentiation medium were split into 15-mL conical polypropylene tubes and centrifuged at 300×*g* for 5 min to precipitate pellets. The caps were loosened, and all pellets acquired a rounded shape by the following day. For chondrogenic differentiation, the medium was supplemented with 10 ng/mL transforming growth factor beta-1 (TGF-β1) (Peprotech Inc., NJ, USA), 100 nM dexamethasone (Sigma-Aldrich), 37.5 μg/mL ascorbic acid (Sigma-Aldrich), 1% insulin-transferrin-selenium (ITS, Roche), and 1 mM sodium pyruvate (Wako) with culturing for 6 weeks. The cultured 3D pellet were fixed with 10% formalin solution for 10 min and dehydrated by sequential addition of 50%, 70%, 90%, 95%, and 100% ethanol for 30 min each. Then, half of the volume of 100% ethanol was removed and replaced with xylene for 30 min. The 3D pellets were incubated in 100% xylene twice for 30 min each and were then embedded in paraffin and sliced into 5-µm thick serial sections. The sections were deparaffinized and rehydrated by three incubation steps in 100% xylene and ethanol for 5 min each. The accumulation of proteoglycan-producing cells was detected by Alcian blue (Sigma-Aldrich).

### Reverse transcription-polymerase chain reaction (RT-PCR)

Total RNA of differentiated and undifferentiated aging ASCs and DFATs cells was extracted from cultured cells using an RNeasy Mini kit (Qiagen, Hilden, Germany). RNA was quantitated by measuring the ratio of absorbance at 260/280 nm. cDNA was synthesized from 1 µg RNA using a High Capacity cDNA Synthesis kit (Applied Biosystems, Carlsbad, CA). A PCR Supermix Platinum kit (Invitrogen) was used with the following thermal cycling conditions: preincubation at 94 °C for 2 min, followed by 35 cycles of denaturation at 94 °C for 30 s (s), primer annealing at 52–60 °C for 30 s and extension at 72 °C for 1 min. Finally, a postextension step was performed at 72 °C for 7 min. The PCR products were electrophoresed on 2% agarose gel (Nippon gene, Japan) and stained with 0.5 µg/mL ethidium bromide (EtBr). Primer sequences used for RT-PCR are listed in supplementary table 1.

### Cellular senescence

Both aging and young ASCs and DFAT cells at P3 and P8 were seeded in 6-well plates for 48 h. A senescence cell histochemical staining kit (SIGMA, Saint Louis, Missouri, USA) was used according to the manufacturer’s specifications. Cellular senescence was scored by visualization using a phase-contrast inverted microscope and represented in blue. The percentage of cellular senescence in all cultures was determined in randomly selected fields and calculated as follow: 100 × positive-stained cells/total number of cells.

### Gene promoter regions

The proximal promoters of four adipogenic genes—PPARγ2, LPL, and FABP4, according to Noer et al. [8]; and C/EBPα, originally designed in this study—were examined. The promoter region of PPARγ2 spanned nucleotides 108–587 and contained six methylated cytosines in CpG dinucleotides (CpGs) within 264 base pairs (bp) upstream from the ATG translation start site. The LPL promoter spanned nucleotides 1321–1777 and contained 11 CpGs within 134 bp upstream of the ATG codon. The FABP4 promoter spanned nucleotides 23,483–23,895 and contained 4 CpGs within 130 bp upstream of the ATG codon, and the C/EBPα promoter spanned nucleotides 34–453 and contained 54 CpGs within 130 bp upstream of the ATG codon.

### DNA extraction and bisulfite modification

Genomic DNA was extracted from P3 and P8 of aging and young ASCs and DFAT cells using a DNeasy® Blood and Tissue kit (Qiagen). In brief, cells cultured in 60-mm^2^ dishes were detached with trypsin and resuspended in 200 μL of PBS. Each sample was digested with 20 μL proteinase K and lysis buffer obtained from the manufacturer. DNA was subjected to bisulfite conversion using an EpiTect® bisulfite kit (Qiagen). In brief, 1 μg of DNA was mixed with the bisulfite mixture, and reactions were performed with the following thermal cycling conditions: three cycles of denaturation at 99 °C and incubation at 60 °C for approximately 5 h each. Bisulfite primers sets were used in this study, as shown in supplementary table 2. PCR was then used to amplify bisulfite-modified DNA with a Supermix Platinum kit under the following condition: preincubation at 94 °C for 2 min; 40 cycles of denaturation at 94 °C for 1 min, primer annealing at 54–57 °C for 1 min, and extension at 65 °C for 1 min; and a postextension step at 65 °C for 7 min.

### Combined bisulfite restriction analysis (COBRA)

For COBRA, PCR products were digested overnight with 20 unit (U) of restriction enzymes specific for the CpG methylated restriction sites. The restriction enzymes, *HpyCH4IV* (ACGT) and *Taq I* (TCGA) (New England Biolabs, Ipswich, MA, USA) can cleave 1 or 2 sites in bisulfite-modified sequences. The digested PCR products were electrophoresed on 2% agarose gel and stained with 0.5 µg/mL EtBr. After enzymatic digestion, each gene was cleaved into 3–6 fragments with different amplicon lengths, as shown in supplementary table 3. The amplicons of PPARγ2 were 62, 181, 237, 299, 418, and 480 bp long. The amplicons of LPL were 121, 164, 172, 285, 336, and 457 bp long. The amplicons of FABP4 were 56, 85, 141, 272, 357, and 413 bp long. The amplicons of C/EBPα were 171, 249, and 420 bp long. The band intensities of the digested bands were measured to determine the relative copy numbers of fragments using MultiGauge V3.0 software (Fujifilm, Japan) and the methylation percentages were calculated with the following formula: methylation percentage = 100 × digested fragments/undigested fragments + digested fragments.

### Statistical analysis

Data are reported as the mean ± standard deviation (SD). Data were tested for normality with the Shapiro–Wilk test. The independent sample *t* test was used to compared two values, and one-way ANOVA was examined when values among ≥ 3 groups. Tukey’s post hoc test was used; however, the Games-Howell test for DNA was used for the C/EBPα DNA methylation analysis due to the data did not meet the homogeneity of variances assumption and the assumption that the mean of residual is 0. Differences for which *P* < 0.05 were considered statistically significant.

## Results

### Early-passage DFAT cells exhibit a morphology similar to that ASCs

**Fig. 1:**
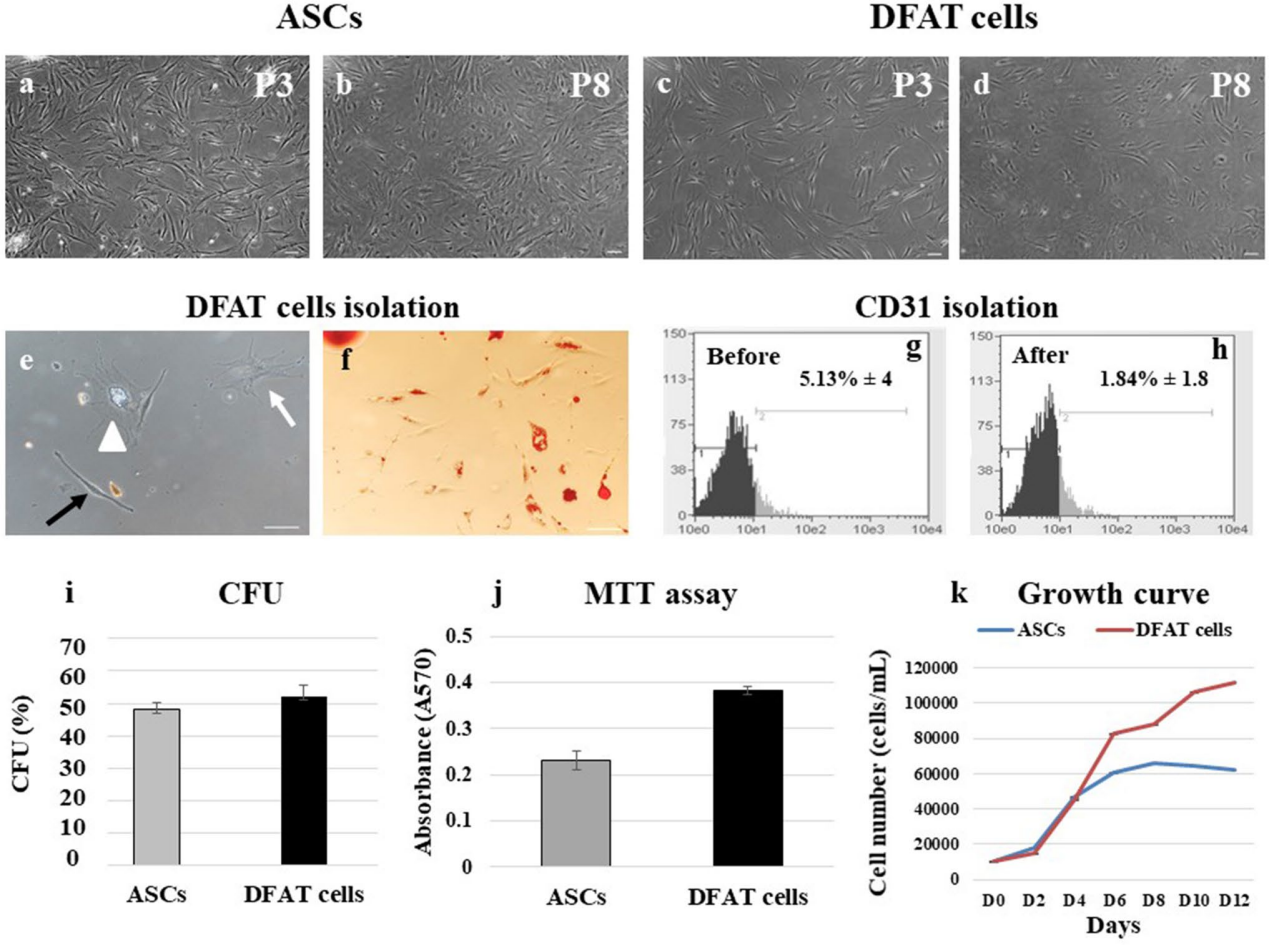
Cell morphology, CFU, MTT and PDT assays. **a**, **b** ASCs at passage 3 (P3) and passage 8 (P8) exhibited a fibroblast-like morphology. **c**, **d** DFAT cells of P3 and P8 also exhibited a fibroblast-like morphology. **e** In primary culture, DFAT cells can dedifferentiate from mature adipocytes and exhibit various morphologies: fibroblast-like (black arrow), polyhedral (white arrow), and lipid droplet-containing (white triangle). **f** Primary cells were stained with Oil Red O. **g**, **h** Via magnetic bead isolation, the percentage of CD31^+^ cells was reduced from 5.13% ± 4 to 1.84% ± 1.8. (*n* = 3).** i** Average %CFU for ASCs and DFAT cells. The proliferation of ASCs and DFAT cells was assessed using MTT (**j**) and PDT (**k**) assays (*n* = 4, each assay). The scale bar represents 100 μm

To compare the phenotypic characteristics of ASCs and DFAT cells, P3 and P8 cells were used as early- and late-passage cells, respectively. Both early- and late-passage ASCs generally demonstrated a spindle shape or fibroblast-like cells morphology (Fig. 1a, b), whereas early-passage DFAT cells exhibited a mostly fibroblast-like morphology but gradually acquired a polyhedral shape by late passages (Fig. 1c, d). Additionally, the primary cultures of DFAT cells demonstrated that DFAT cells could dedifferentiate from mature adipocytes, which have been suggested to have a limited differentiation capacity, to various cell phenotypes, including fibroblast-like cells, polyhedral cells, and cells containing lipid droplets (Fig. 1e). Positive Oil Red O staining was observed in cells containing lipid droplets from DFAT cells primary cultures (Fig. 1f).

### Purification of heterogeneous ASCs

For purification of heterogeneous ASCs, erythrocyte lysis buffer and endothelial cell exclusion methods were used with magnetic bead isolation procedures. ASCs with CD31 expression were discarded, and the amount of CD31 before and after isolation in primary cultured ASCs was measured by flow cytometry. The percentages of CD31^+^ASCs before and after isolation were 5.13% ± 4 and 1.84% ± 1.8, respectively (Fig. 1g, h, *P* = *0.85*).

### Colony-forming ability

The colonies formed by aging ASCs and DFAT cells showed no morphological differences. DFAT cells exhibited a higher CFU, (51.92 ± 3.56%) than ASCs (48 ± 2.4%) (Fig. 1i); however, the difference was not significant (*P* = 0.53).

### Aging DFAT cells demonstrate higher proliferative function than ASCs

To compare the proliferative ability of aging ASCs and DFAT cells, MTT assay, growth curve and PDT assay were used. DFAT cells demonstrated significantly higher absorbance values indicating viable cells (0.382 ± 0.01) than ASCs (0.231 ± 0.21) and control cells (0.07 ± 0.02) in the MTT assay (Fig. 1j), but the differences among the three groups were nonsignificant (*P* = 0.73). The growth curve of DFAT cells showed the following mean numbers of cells from day (D) 0 to D12: D0 (1000 ± 0), D2 (14,861.11 ± 3.5), D4 (45,416.67 ± 18.72), D6 (82,500 ± 11.82), D8 (87,916.67 ± 33.12), D10 (106,666.7 ± 33.67), and D12 (111,805.6 ± 25.14). For ASCs, the corresponding cell counts were: D0 (1000 ± 0), D2 (18,194.44 ± 4.6), D4 (46,805.56 ± 10.56), D6 (60,694.44 ± 10.88), D8 (66,250 ± 5.71), D10 (64,160.7 ± 9.15), and D12 (61,800.5 ± 14.6). For the PDT assay, data were taken from the linear part of the curve and demonstrated a slightly shorter PDT (38.82 h) DFAT cells than in ASCs (55.23 h) (Fig. 1k).

### Aging ASCs and DFAT cells express similar cell surface markers

**Fig. 2:**
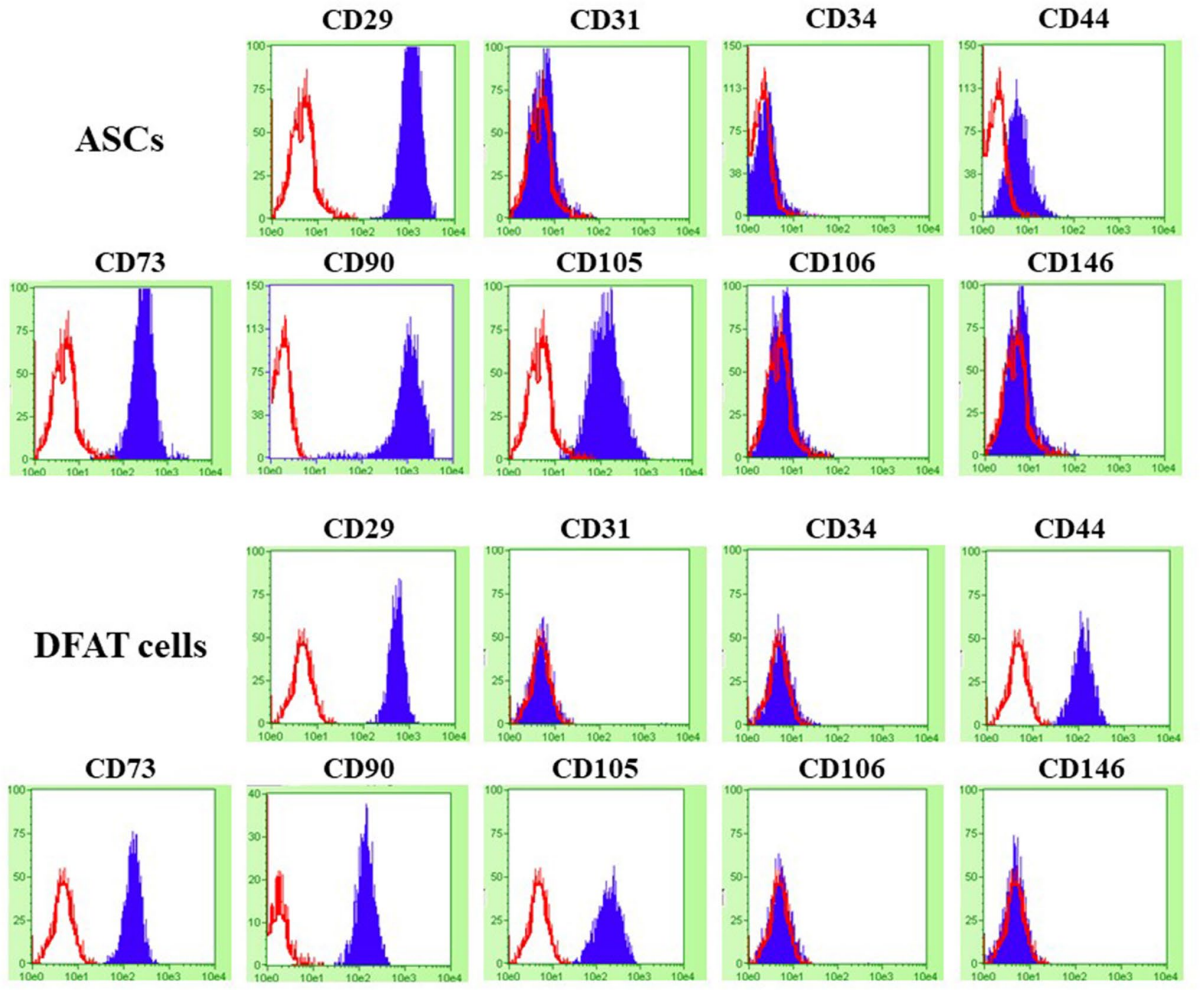
Histograms of the flow cytometric analysis results in ASCs and DFAT cells for analyzing the surface antigen markers for the mesenchymal lineage (CD29, CD44, CD73, CD90, CD105) and the hematopoietic or perivascular lineage (CD34, CD106, CD146), along with an endothelial marker (CD31). Negative control in each sample has been depicted in the red outlines, and target surface antigen markers were shown in the blue area

Representative flow cytometric histograms are shown in Fig. 2. Both ASCs and DFAT cells uniformly expressed the cell surface markers CD29 (Integrin β1, fibronectin receptor), mesenchymal stromal cell markers (CD44, CD73, CD90 and CD105) in up to 90% of cells, except in CD44 of ASCs and CD90 of DFAT cells were shown the expression approximately 70% and 83% of cells, respectively. All cells did not express hematopoietic stem cells, endothelial cells, and perivascular cells markers (CD31, CD34, CD106, CD146).

### Immunofluorescence characterizations

**Fig. 3:**
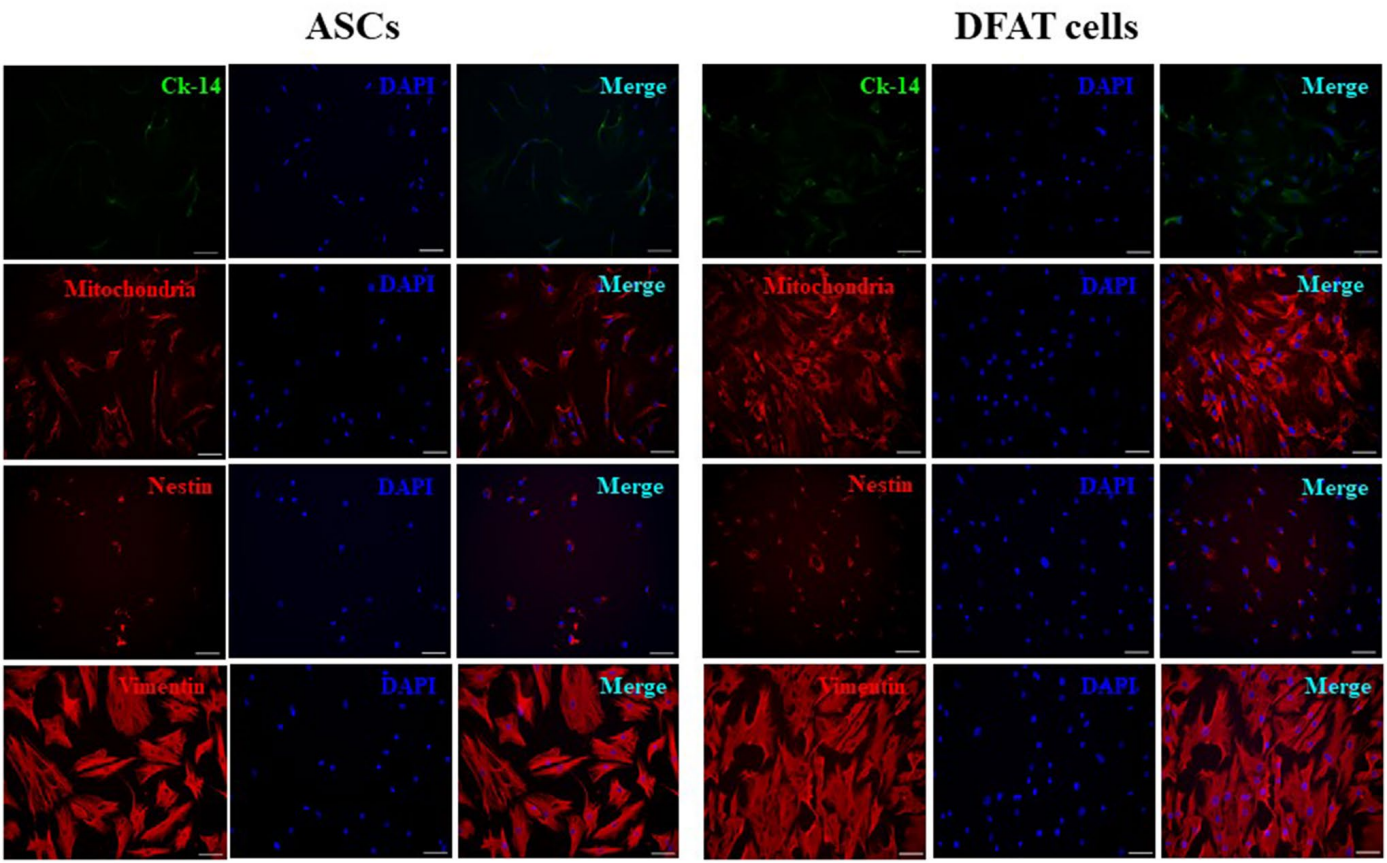
Immunofluorescence characterization of the markers cytokeratin-14 (Ck-14), mitochondria, nestin, and vimentin. Immunofluorescence indicated positive expression of mitochondria, nestin, and vimentin but not Ck-14 in ASCs and DFAT cells. DAPI (blue) was used to stain nuclei. The scale bar represents 100 μm

**Fig. 4:**
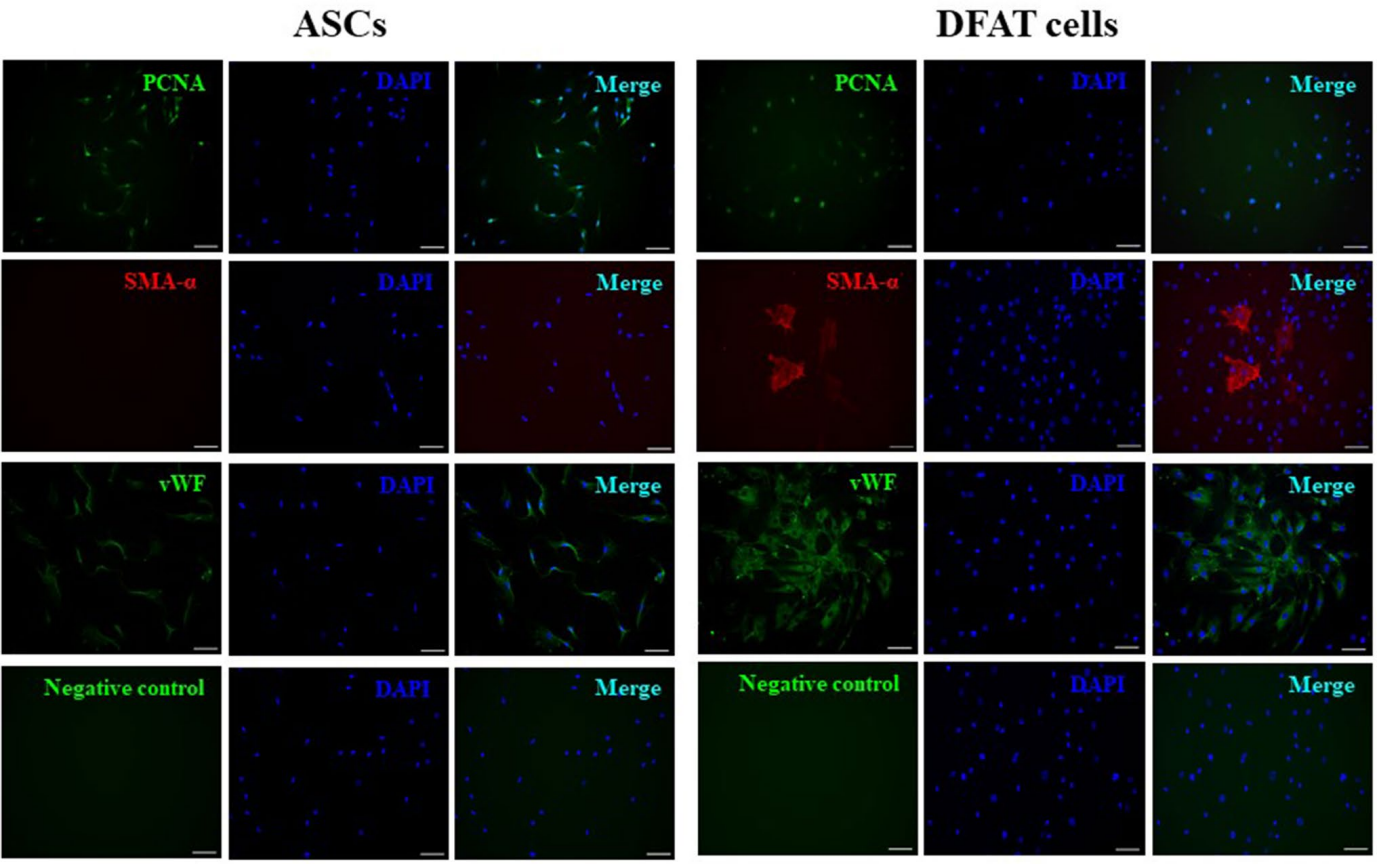
Immunofluorescence characterization of the markers PCNA, SMA-α and vWF. Immunofluorescence indicated strong expression of PCNA, SMA-α and vWF in DFAT cells. DAPI (blue) was used to stain nuclei. The scale bar represents 100 μm

Aging ASCs and DFAT cells were negative for expression of CK-14, which labels the basal layer of stratifying squamous and nonsquamous epithelial cells. However, all cells were positive for mitochondria, nestin and vimentin staining, demonstrating their functional MSCs characteristics (Fig. 3). Interestingly, PCNA, the marker for DNA replication in cell division, and vWF, an endothelial cell marker, were also positively expressed in both ASCs and DFAT cells. The SMA-α marker was rarely detected in DFAT cells but was completely absent from ASCs (Fig. 4).

### ASCs and DFAT cells exhibit multilineage differentiation

**Fig. 5:**
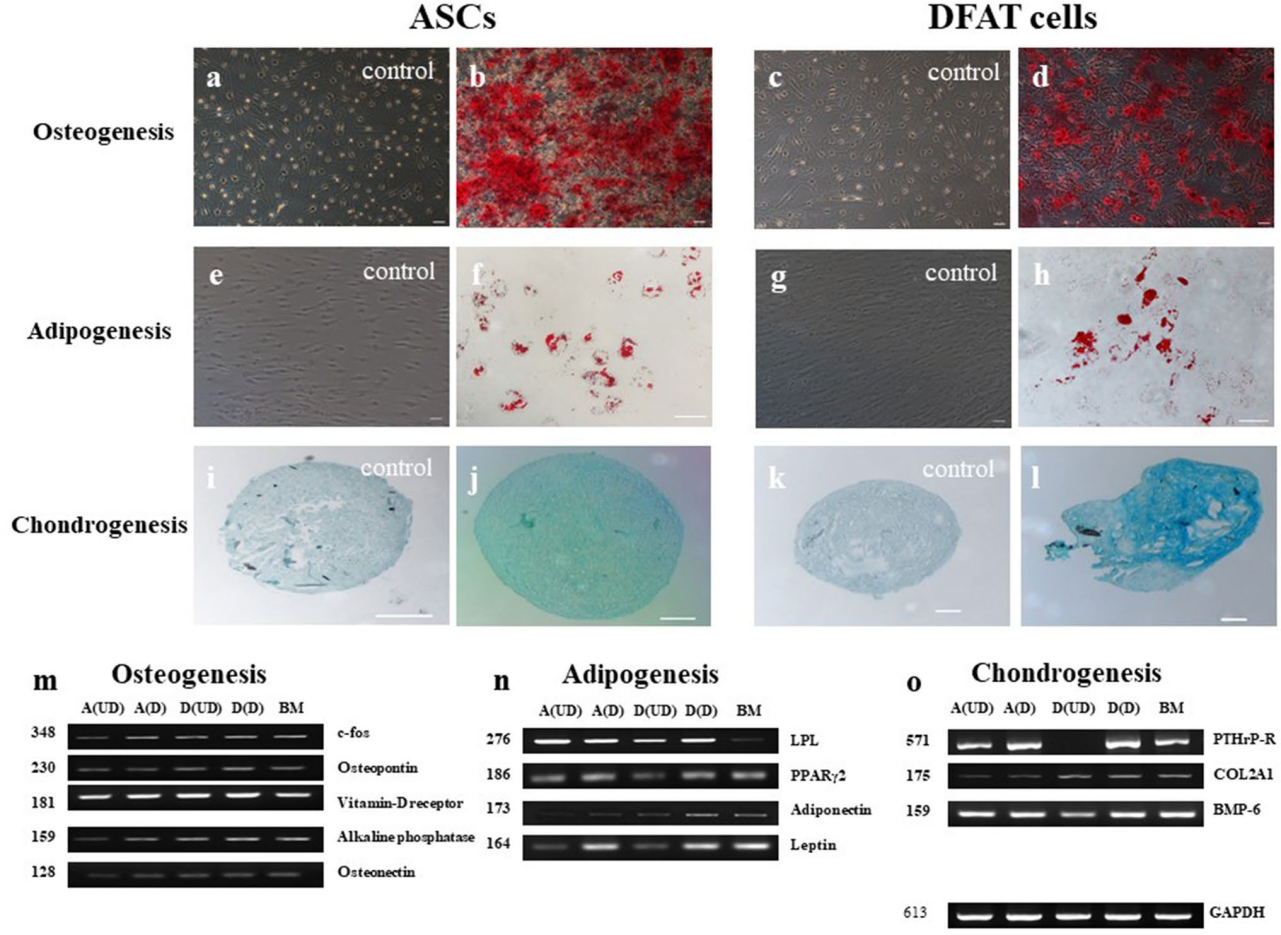
Multilineage differentiation potential and gene expression patterns. **a**–**d** Osteogenic differentiation of ASCs and DFAT was confirmed by Alizarin Red staining. **e**–**h** Adipogenic differentiation was confirmed by Oil Red O staining of lipid droplets. **i–l** 3-D pellets of chondrogenically differentiated cells were stained with Alcian blue. **m–o** Gene expression in each lineage was compared among undifferentiated ASCs (A(UD)), differentiated ASCs (A(D)), undifferentiated DFAT cells (D(UD)), differentiated DFAT cells (D(D)), and BMMSCs (BM). GAPDH was used as the endogenous control gene. The scale bar represents 100 μm

Multilineage differentiation via osteogenesis, adipogenesis, and chondrogenesis was compared in aging ASCs and DFAT cells. Both cell types exhibited similar patterns in all differentiation lineages. In osteogenically differentiated cells, calcified matrix aggregations (stained with Alizarin Red) were primarily detected at 2 weeks, but we continuously induced ASCs (Fig. 5a, b) and DFAT cells (Fig. 5c, d) for 3 weeks. Under suboptimal conditions, ASCs (Fig. 5e, f) and DFAT cells (Fig. 5g, h) preferentially underwent adipogenic differentiation, as shown by high production of lipid droplets, which were stained with Oil Red O, in 14–17 days. After the differentiation period, chondrogenic differentiation resulted in production of proteoglycans, which were stained with Alcian blue: control of ASCs and DFAT cells (Fig. 5i, k, respectively), chondrogenic differentiation of ASCs and DFAT cells (Fig. 5j, l, respectively). All multilineage differentiation was confirmed by RT-PCR assessment of gene expression compared to that in undifferentiated cells and BMMSCs (Fig. 5m–o).

### A low percentage of late-passage DFAT cells exhibit cellular senescence

**Fig. 6:**
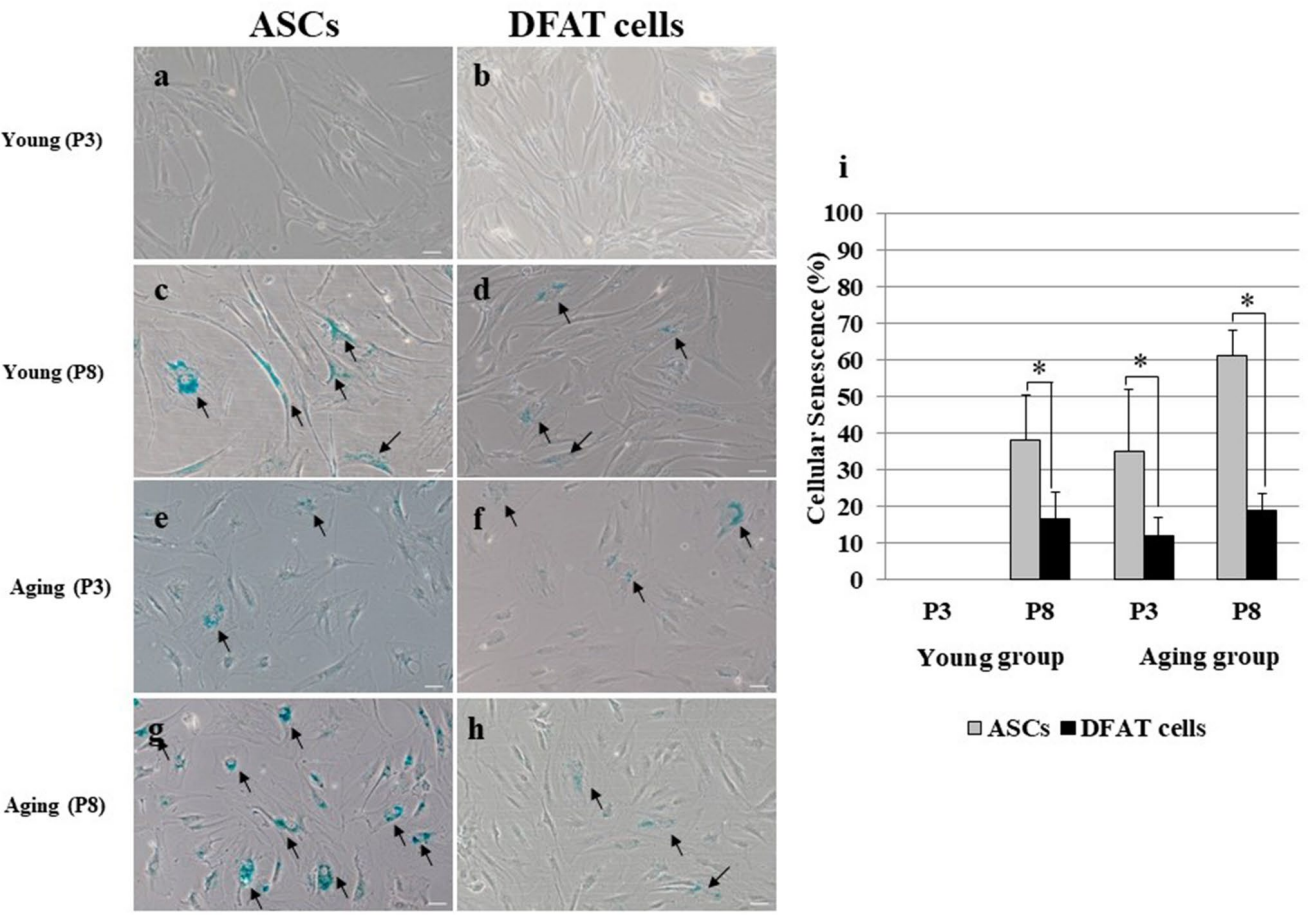
Comparison of cellular senescence in early- and late-passage cells (*n* = 3). **a**, **b** P3 ASCs and DFAT cells from the young group. **c**, **d** P8 ASCs and DFAT cells from the young group. **e**, **f** P3 ASCs and DFAT cells from the aged group. **g**, **h** P8 ASCs and DFAT cells from the young group. **i** Percent of senescent cells. The percent of senescent ASCs was significantly higher than that of senescent DFAT cells at all passages (*n* = 3, *P < 0.05). The scale bar represents 100 μm

Cellular senescence was assessed by measuring the activity of β-galactosidase (β-gal), which is expressed in cells during senescence. No early-passage cells from subjects in the young group exhibited cellular senescence (Fig. 6a, b). ASCs from both young and aging subjects demonstrated higher level of cellular senescence than DFAT cells (Fig. 6c–h). In addition, ASCs at P8 showed significantly higher level of cellular senescence than DFAT cells in the young group (*P* = 0.001, Fig. 6i). Moreover, ASCs in the aging group showed similar levels of cellular senescence. Aging ASCs at P3 had significantly higher level of senescence than DFAT cells at P3 (35.05 ± 16.88 and 12.11 ± 5.05, *P* = 0.00). At P8, cellular senescence was increased in ASCs (60.97 ± 7.28), but remained low (18.78 ± 4.9, *P* = 0.02) in DFAT cells (Fig. 6i).

### Adipogenic genes demonstrate hypermethylation except in C/EBPα gene

**Fig. 7:**
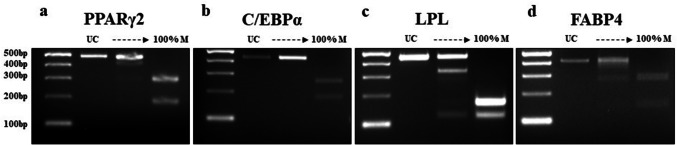
DNA methylation profiling of all adipogenic genes by COBRA. All PCR products were analyzed by COBRA with restriction enzymes: *HpyCH4IV* (ACGT) for PPARγ2, C/EBPα, and LPL and *Taq I* (TCGA) for FABP4. The lengths of all gene fragments after enzymatic digestion were as follows: **a** PPARγ2: 62, 181, 237, 299, 418, and 480 bp (480 bp was seen in the undigested product); **b** C/EBPα: 171, 249, and 420 bp (420 bp was seen in the undigested product); **c** LPL: 121, 164, 172, 285, 336, and 457 bp (457 bp was seen in the undigested product); and **d** FABP4: 56, 85, 141, 272, 357, and 413 bp (413 bp was seen in the undigested product)

**Fig. 8:**
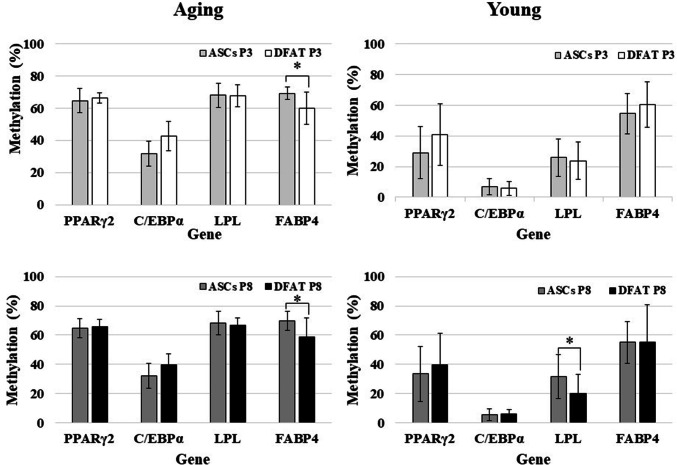
Comparison of DNA methylation profiles between ASCs and DFAT cells [aged group (*n* = 21), and young group (*n* = 25)]

PCR amplicons of each digested and undigested fragment of four adipogenic genes are shown in Fig. 7. Regarding the methylation status of genes in ASCs and DFAT cells, in cultured P3 cells from the aging group, three genes—PPARγ2, LPL, and FABP4 in ASCs (64.64 ± 7.4, 67.96 ± 7.48, 69.28 ± 3.86) and DFAT cells (66.4 ± 3.16, 67.64 ± 6.98, 59.76 ± 10.06)—demonstrated hypermethylation. Statistically significant differences were found only for FABP4, which indicated that ASCs have higher hypermethylation frequency than DFAT cells at both P3 (ASCs, 69.28 ± 3.86; DFAT cells, 59.76 ± 10.06, *P* = 0.011) and P8 (ASCs, 69.64 ± 6.61; DFAT cells, 58.44 ± 13.15, *P* = 0.024). Moreover, LPL gene methylation in P8 cells from the young group differed significantly between ASCs (31.48 ± 15.29) and DFAT cells (20.03 ± 13.30, *P* = 0.02) (Fig. 8). On the other hand, C/EBPα demonstrated hypomethylation (a methylation rate of less than 50%) in ASCs and DFAT cells from both the aging and young group (Fig. 8). In cultured cells from aging group at P8, 3 genes—PPARγ2, LPL, and FABP4 in ASCs (64.57 ± 6.62, 68.07 ± 8.02, 69.64 ± 6.61) and DFAT cells (65.72 ± 4.94, 66.5 ± 5.28, 58.44 ± 13.15)—demonstrated hypermethylation (Fig. 8).

**Fig. 9:**
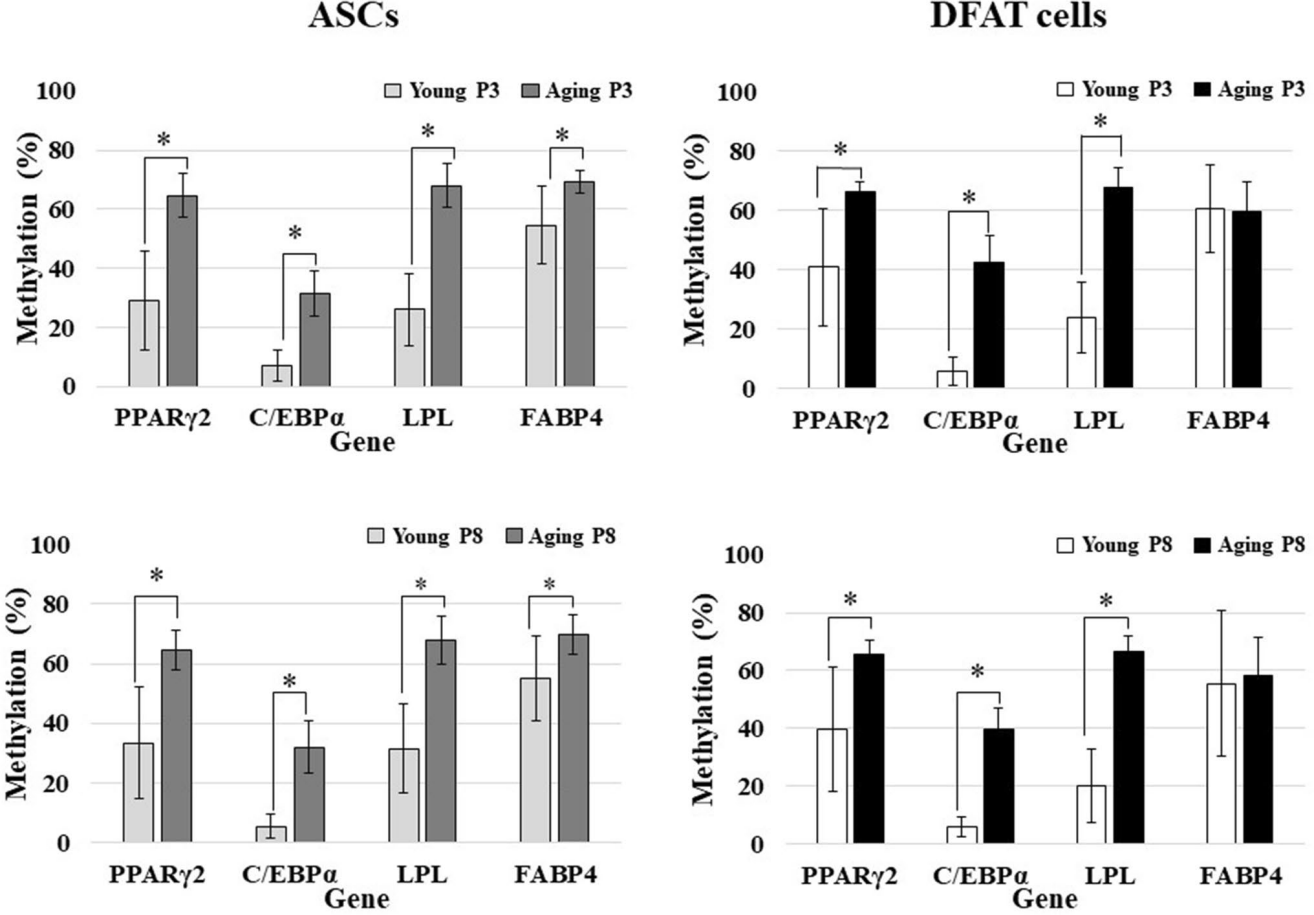
Comparison of DNA methylation profiles between the aged and young groups [aged group (*n* = 21), and young group (*n* = 25)]

**Fig. 10:**
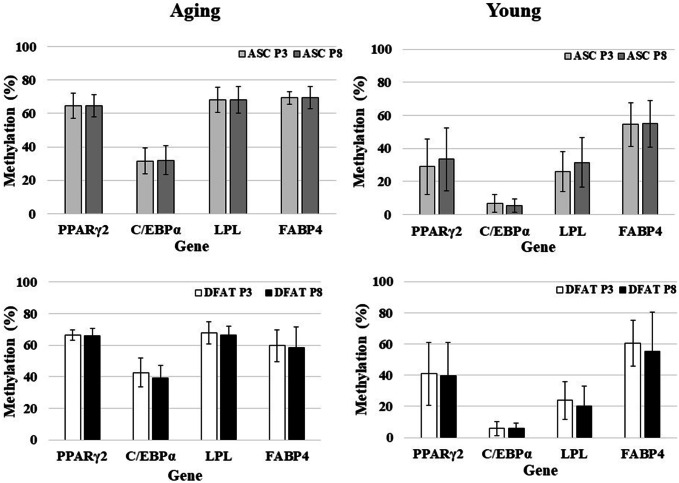
Comparison of DNA methylation profiles between passage 3 (P3) and passage 8 (P8) cells [aged group (*n* = 21), and young group (*n* = 25)]

Comparison of the methylation status between the aging and young groups revealed that these adipogenic genes demonstrated a similar pattern in both ASCs and DFAT cells: hypermethylation in the aging group and hypomethylation in the young group. The difference between the aging and young groups in all genes and for all passages were statistically significant (*P* < 0.05, Fig. 9). However, the methylation patterns observed in P3 and P8 cells did not differ significantly between either passage of ASCs and DFAT cells (*P* = 0.71 and *P* = 0.85, for ASCs and DFAT cells, respectively; Fig. 10).

## Discussion

ASCs and DFAT cells have emerged for use in tissue engineering and regeneration. Although several studies have demonstrated the utility of ASCs and DFAT cells, a comparison of methylation between these cells has not been performed. Our findings demonstrated that the methylation profile patterns of DFAT cells are similar to those of ASCs, with hypermethylation observed in the matched subject groups. Hypermethylation was detected in three adipogenic genes: PPARγ2, LPL, and FABP4. These genes exhibited the same pattern of methylation profiles, which were maintained from early- to late-passages. These results are consistent with those of Jones and Takai, who demonstrated hypermethylation of an adipogenic gene by bisulfite sequencing [21], but conflict with other groups’ reports of DNA and histone hypomethylation [8, 14, 32, 39]. In these previous studies, ASCs from young subjects were investigated, and the mosaic bisulfite sequencing method was used, whereas we used COBRA in the present study. However, these studies indicated that the DNA methylation status is reflected as ASCs can maintain the stability of the cells via epigenetics regulations. Even in self clones, fresh, uncultured, differentiated, early-passage, late-passage and senescence-stage ASCs have been verified to have nearly equal DNA methylation percentages [8, 32], corroborated our findings. Thus, adipose cells may be attributed to direct molecular modification in epigenetics rather than to changes in cell number, proliferation, adipogenic gene expression, and adipogenic differentiation.

The hypermethylation status in our study might have resulted from limitations inherent to our method. We used COBRA, which is faster than bisulfite sequencing and allows appropriate screening method in large samples. However, the specific restriction enzymes used and the positions of the CpG in each gene might be used to identify inconsistencies. In addition, the methylation status depends on the CpG content in the promoter region. Promoters can be grouped into three classes: low, intermediate, and high CpG content promoters [40, 41]. Here, the adipogenic genes PPARγ2 (6 CpGs), FABP4 (3 CpGs), and LPL (11 CpGs) were classified as low CpG content promoters, but C/EBPα (52 CpGs) was classified as a high CpG content promoter. High CpG content promoters are strongly associated with a non-methylated or weakly methylated status. Thus, the hypomethylation of C/EBPα gene indicated that it was the least methylated gene in this study. To address these limitations driven by the gene promoter characteristics and restriction enzyme digestion, other methods such as bisulfite sequencing, methylation-specific PCR (MSP), or pyrosequencing should be performed with the C/EBPα gene.

The adipogenic genes investigated in our study were selected by their important roles in the adipogenic processes and their microarray data, which were examined in our ASCs from female aging subject and revealed relative mRNA expression level (unpublished data). Gene profiling showed that the relative mRNA expression levels of PPARγ2, C/EBPα, LPL, and FABP4 were upregulated approximately 8-, 10-, 6-, and 6-folds, respectively, in this subject compared with BMMSCs. In this study, we selected PPARγ2 and C/EBPα, the critical factors for adipogenic differentiation. These genes can be detected immediately after differentiation and maintained for prolonged periods until lipid droplet formation. Moreover, these two factors are believed to synergistically enhance each other’s participation in adipogenic differentiation and are necessary for induction [42, 43]. A recent study reported that PPARγ2 was suitable for DNA methylation analysis because it was suggested to be strongly regulated by DNA methylation [44]. Regarding the LPL gene, in cultured 3T3-preadipocytes, LPL expression is an early marker of adipose differentiation, which can be enhanced by cell–cell contact [45]. FABP4 exhibits different functions; it is a member of the fatty acid-binding protein family that binds fatty acids in the intercellular cavity. FABP4 has an important function in accelerating these binding processes not only intracellular but also in the extracellular membrane [46].

Heterogeneities in ASCs derived from SVF usually result from contamination with endothelial and hematopoietic cells. Although several reports in addition to ours exhibit less contamination by CD31^−^ expressing cells, magnetic bead isolation might provide purer ASCs of mesenchymal cell lineage. Moreover, CD34, a hematopoietic stem cell marker, gradually disappears during the passage of uncultured ASCs at P4 [14]. Our study also demonstrated that CD34 was completely undetected at P4 indicating the homogeneity of ASCs and DFAT cells. In addition, CD106 is commonly detected in BMMSCs in both small blood vessels and the endothelial cell; however, we did not detect this marker. In contrast to other findings, expression of the pericyte marker CD146 was not found in our study, which might attenuate the regenerative potential [47]. Also, CD44 of DFAT cells demonstrated higher positive cells staining than ASCs, which approximately 1.28 times. This result may be suggested that DFAT cells are more homogeneous source than ASCs. Thus, we confirmed that the characteristics of DFAT cells are similar to those of MSCs, including ASCs and BMMSCs [16, 17].

Many theories, such as oxidative stress and proinflammatory cytokines, which subsequently diminish proliferation and differentiation and gradually induce hypermethylation, have been proposed to explain the effects of aging on MSCs [25, 28, 48–50]. Our results demonstrated that DFAT cells derived from aging subjects exhibit robust proliferation and differentiation in early passage, even though they showed DNA hypermethylation. These findings suggest that DNA methylation might be involved in in vivo aging but not in in vitro aging. The correlation between hypermethylation and aging was recently proposed considering the results of cytosine methylation analysis indicating genome-wide and locus-specific differences [25, 32, 33]. A study reported that hypermethylation gradually increased with age of liver tissue but did not significantly change with age in adipose tissue, implying a relationship between hypermethylation and aging and suggesting that hypermethylation acts as a tissue-specific determinant of pathogenesis in adipose tissue. Interestingly, our DNA methylation data demonstrated that aging processes were similar percentages in ASCs and DFAT cells from P3 and P8, which demonstrated a stable pattern from P3 to P8. Importantly, these data suggest that DFAT cells are useful as MSCs given their stability and cellular maintenance.

## Conclusions

ASCs and DFAT cells displayed multilineage differentiation and immunophenotype characteristics similar to those of MSCs. Moreover, they exhibited DNA hypermethylation patterns, indicating a possible relation to in vivo aging. However, these cells can remain stable from early-to late-passages, as indicated by epigenetic patterns. Thus, ASCs and DFAT cells are attractive as alternative cells for use in tissue engineering and regeneration during aging to maintain adipogenic stability and treat other aged-related diseases.

## Electronic supplementary material

Below is the link to the electronic supplementary material.

Supplementary material 1 (JPEG 104 kb)

Supplementary material 2 (JPEG 49 kb)

Supplementary material 3 (JPEG 53 kb)

## References

[1] Gimble JM, Katz AJ, Bunnell BA (2007). Adipose-derived stem cells for regenerative medicine. Circ Res.

[2] Schäffler A, Büchler C (2007). Concise review: adipose tissue-derived stromal cells-basic and clinical implications for novel cell-based therapies. Stem Cells.

[3] Zuk PA, Zhu M, Mizuno H, Huang J, Futrell JW, Katz AJ (2001). Multilineage cells from human adipose tissue: implications for cell-based therapies. Tissue Eng.

[4] Yoshimura K, Shigeura T, Matsumoto D, Sato T, Takaki Y, Aiba-Kojima E (2006). Characterization of freshly isolated and cultured cells derived from the fatty and fluid portions of liposuction aspirates. J Cell Physiol.

[5] Bunnell BA, Estes BT, Guilak F, Gimble JM (2008). Differentiation of adipose stem cells. Methods Mol Biol.

[6] Dmitrieva RI, Minullina IR, Bilibina AA, Tarasova OV, Anisimov SV, Zaritskey AY (2012). Bone marrow- and subcutaneous adipose tissue-derived mesenchymal stem cells differences and similarities. Cell Cycle.

[7] Jin HJ, Bae YK, Kim M, Kwon SJ, Jeon HB, Choi SJ (2013). Comparative analysis of human mesenchymal stem cells from bone marrow, adipose tissue, and umbilical cord blood as sources of cell therapy. Int J Mol Sci.

[8] Noer A, Sørensen AL, Boquest AC, Collas P (2006). Stable CpG hypomethylation of adipogenic promoters in freshly isolated, cultured, and differentiated mesenchymal stem cells from adipose tissue. Mol Biol Cell.

[9] Baglioni S, Francalanci M, Squecco R, Lombardi A, Cantini G, Angeli R (2009). Characterization of human adult stem-cell populations isolated from visceral and subcutaneous adipose tissue. FASEB J.

[10] Prunet-Marcassus B, Cousin B, Caton D, André M, Pénicaud L, Casteilla L (2006). From heterogeneity to plasticity in adipose tissues: site-specific differences. Exp Cell Res.

[11] Zimmerlin L, Donnenberg VS, Pfeifer ME, Meyer EM, Péault B, Rubin JP (2010). Stromal vascular progenitors in adult human adipose tissue. Cytometry A.

[12] Gronthos S, Franklin DM, Leddy HA, Robey PG, Storms RW, Gimble JM (2001). Surface protein characterization of human adipose tissue-derived stromal cells. J Cell Physiol.

[13] Zuk PA, Zhu M, Ashjian P, De Ugarte DA, Huang JI, Mizuno H (2002). Human adipose tissue is a source of multipotent stem cells. Mol Biol Cell.

[14] Boquest AC, Shahdadfar A, Frønsdal K, Sigurjonsson O, Tunheim SH, Collas P (2005). Isolation and transcription profiling of purified uncultured human stromal stem cells: alteration of gene expression after in vitro cell culture. Mol Biol Cell.

[15] Maumus M, Peyrafitte JA, D'Angelo R, Fournier-Wirth C, Bouloumié A, Casteilla L (2011). Native human adipose stromal cells: localization, morphology and phenotype. Int J Obes.

[16] Matsumoto T, Kano K, Kondo D, Fukuda N, Iribe Y, Tanaka N (2008). Mature adipocyte-derived dedifferentiated fat cells exhibit multilineage potential. J Cell Physiol.

[17] Poloni A, Maurizi G, Leoni P, Serrani F, Mancini S, Frontini A (2012). Human dedifferentiated adipocytes show similar properties to bone marrow-derived mesenchymal stem cells. Stem Cells.

[18] Oki Y, Watanabe S, Endo T, Kano K (2008). Mature adipocyte-derived dedifferentiated fat cells can trans-differentiate into osteoblasts in vitro and in vivo only by all-trans retinoic acid. Cell Struct Funct.

[19] Jumabay M, Matsumoto T, Yokoyama S, Kano K, Kusumi Y, Masuko T (2009). Dedifferentiated fat cells convert to cardiomyocyte phenotype and repair infarcted cardiac tissue in rats. J Mol Cell Cardiol.

[20] Obinata D, Matsumoto T, Ikado Y, Sakuma T, Kano K, Fukuda N (2011). Transplantation of mature adipocyte-derived dedifferentiated fat (DFAT) cells improves urethral sphincter contractility in a rat model. Int J Urol.

[21] Jones PA, Takai D (2001). The role of DNA methylation in mammalian epigenetics. Science.

[22] Bernstein BE, Meissner A, Lander ES (2007). The mammalian epigenome. Cell.

[23] Boquest AC, Noer A, Collas P (2006). Epigenetic programming of mesenchymal stern cells from human adipose tissue. Stem Cell Rev.

[24] Collas P (2009). Epigenetic states in stem cells. Biochim Biophys Acta.

[25] Thompson RF, Atzmon G, Gheorghe C, Liang HQ, Lowes C, Greally JM (2010). Tissue-specific dysregulation of DNA methylation in aging. Aging Cell.

[26] Pollina EA, Brunet A (2011). Epigenetic regulation of aging stem cells. Oncogene.

[27] Teschendorff AE, Menon U, Gentry-Maharaj A, Ramus SJ, Weisenberger DJ, Shen H (2010). Age-dependent DNA methylation of genes that are suppressed in stem cells is a hallmark of cancer. Genome Res.

[28] Kim M, Kim C, Choi YS, Kim M, Park C, Suh Y (2012). Age-related alterations in mesenchymal stem cells related to shift in differentiation from osteogenic to adipogenic potential: implication to age-associated bone diseases and defects. Mech Ageing Dev.

[29] Harman D (2003). The free radical theory of aging. Antioxid Redox Signal.

[30] Cadenas E, Davies KJ (2000). Mitochondrial free radical generation, oxidative stress, and aging. Free Radic Biol Med.

[31] Chung HY, Kim HJ, Kim JW, Yu BP (2001). The inflammation hypothesis of aging: molecular modulation by calorie restriction. Ann NY Acad Sci.

[32] Noer A, Boquest AC, Collas P (2007). Dynamics of adipogenic promoter DNA methylation during clonal culture of human adipose stem cells to senescence. BMC Cell Biol.

[33] van den Dungen MW, Murk AJ, Kok DE, Steegenga WT (2016). Comprehensive DNA methylation and gene expression profiling in differentiating human adipocytes. J Cell Biochem.

[34] Day K, Waite LL, Thalacker-Mercer A, West A, Bamman MM, Brooks JD (2013). Differential DNA methylation with age displays both common and dynamic features across human tissues that are influenced by CpG landscape. Genome Biol.

[35] Rönn T, Volkov P, Gillberg L, Kokosar M, Perfilyev A, Jacobsen AL (2015). Impact of age, BMI and HbA1c levels on the genome-wide DNA methylation and mRNA expression patterns in human adipose tissue and identification of epigenetic biomarkers in blood. Hum Mol Genet.

[36] Liu GH, Ding Z, Izpisua Belmonte JC (2012). iPSC technology to study human aging and aging-related disorders. Curr Opin Cell Biol.

[37] Kornicka K, Marycz K, Marędziak M, Tomaszewski KA, Nicpoń J (2017). The effects of the DNA methyltranfserases inhibitor 5-Azacitidine on ageing, oxidative stress and DNA methylation of adipose derived stem cells. J Cell Mol Med.

[38] Guan JS, Haggarty SJ, Giacometti E, Dannenberg JH, Joseph N, Gao J (2009). HDAC2 negatively regulates memory formation and synaptic plasticity. Nature.

[39] Sørensen AL, Jacobsen BM, Reiner AH, Andersen IS, Collas P (2010). Promoter DNA methylation patterns of differentiated cells are largely programmed at the progenitor stage. Mol Biol Cell.

[40] Takai D, Jones PA (2002). Comprehensive analysis of CpG islands in human chromosomes 21 and 22. Proc Natl Acad Sci USA.

[41] Weber M, Hellmann I, Stadler MB, Ramos L, Pääbo S, Rebhan M (2007). Distribution, silencing potential and evolutionary impact of promoter DNA methylation in the human genome. Nat Genet.

[42] Hamm JK, Park BH, Farmer SR (2001). A role for C/EBPbeta in regulating peroxisome proliferator-activated receptor gamma activity during adipogenesis in 3T3-L1 preadipocytes. J Biol Chem.

[43] Avram MM, Avram AS, James WD (2007). Subcutaneous fat in normal and diseased states 3. Adipogenesis: from stem cell to fat cell. J Am Acad Dermatol.

[44] Fujiki K, Kano F, Shiota K, Murata M (2009). Expression of the peroxisome proliferator activated receptor gamma gene is repressed by DNA methylation in visceral adipose tissue of mouse models of diabetes. BMC Biol.

[45] Maeda K, Uysal KT, Makowski L, Görgün CZ, Atsumi G, Parker RA (2003). Role of the fatty acid binding protein mal1 in obesity and insulin resistance. Diabetes.

[46] Zimmerlin L, Donnenberg VS, Rubin JP, Donnenberg AD (2013). Mesenchymal markers on human adipose stem/progenitor cells. Cytometry A.

[47] Kirkland JL, Tchkonia T, Pirtskhalava T, Han J, Karagiannides I (2002). Adipogenesis and aging: does aging make fat go MAD?. Exp Gerontol.

[48] Van Zant G, Liang Y (2003). The role of stem cells in aging. Exp Hematol.

[49] Permana PA, Menge C, Reaven PD (2006). Macrophage-secreted factors induce adipocyte inflammation and insulin resistance. Biochem Biophys Res Commun.

[50] Suganami T, Tanimoto-Koyama K, Nishida J, Itoh M, Yuan X, Mizuarai S (2007). Role of the Toll-like receptor 4/NF-kappaB pathway in saturated fatty acid-induced inflammatory changes in the interaction between adipocytes and macrophages. Arterioscler Thromb Vasc Biol.

