# Supplementary figures and images for: Characterization and comparative DNA methylation profiling of four adipogenic genes in adipose-derived stem cells and dedifferentiated fat cells from aging subjects
Source: Hum Cell. 2020 Jun 3;33(4):974–89. doi: 10.1007/s13577-020-00379-x (PMC7505878; doi:10.1007/s13577-020-00379-x)

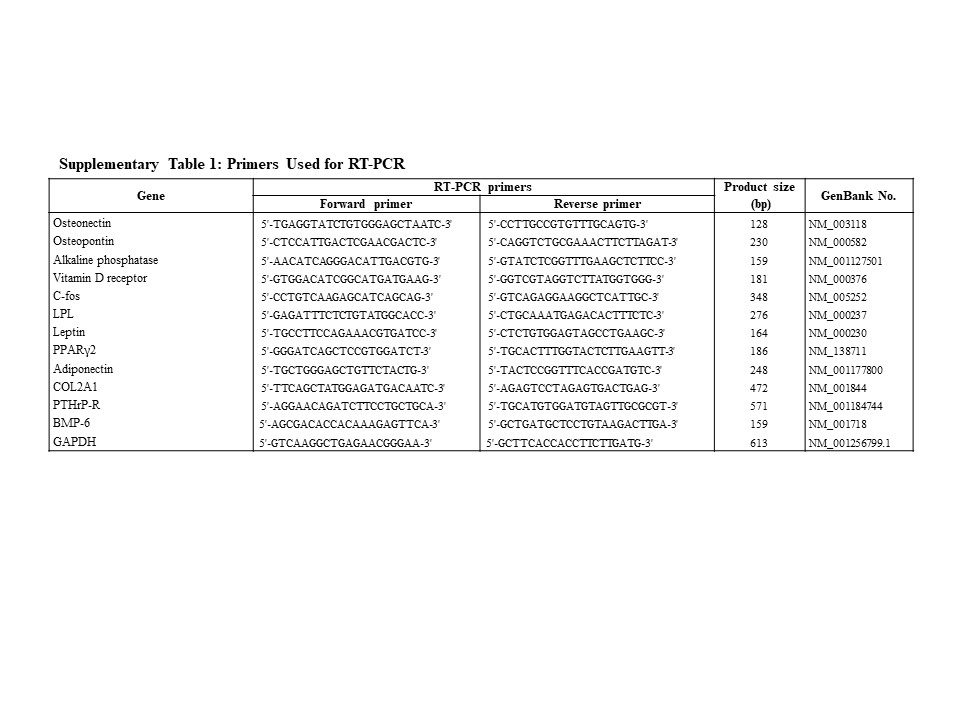

Supplement: Supplementary file 1 — Supplementary material 1 (JPEG 104 kb) [file 13577_2020_379_MOESM1_ESM.jpg]

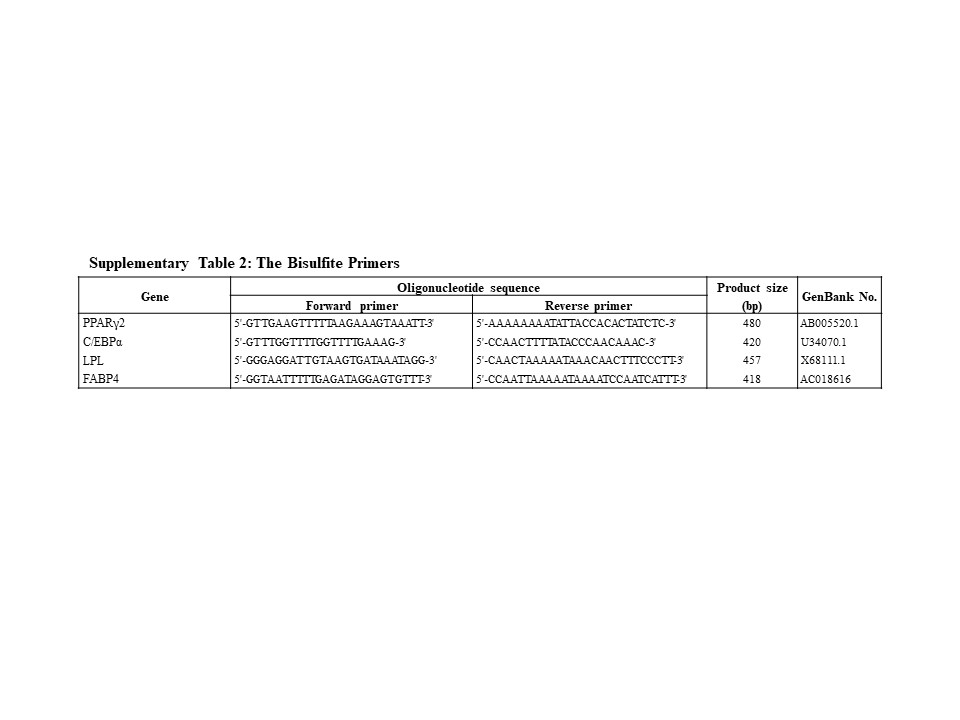

Supplement: Supplementary file 2 — Supplementary material 2 (JPEG 49 kb) [file 13577_2020_379_MOESM2_ESM.jpg]

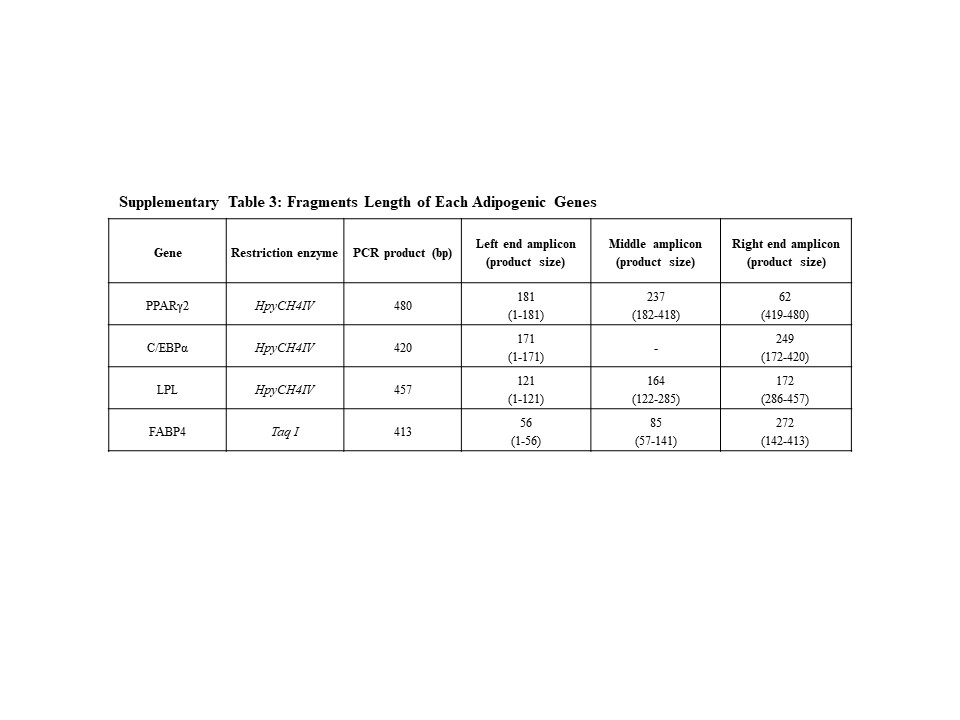

Supplement: Supplementary file 3 — Supplementary material 3 (JPEG 53 kb) [file 13577_2020_379_MOESM3_ESM.jpg]
